# Veterinary Prescriptions of Antibiotics Approved for Human Use: A Five-Year Analysis of Companion Animal Use and Regulatory Gaps in Brazil

**DOI:** 10.3390/vetsci12070652

**Published:** 2025-07-09

**Authors:** Rana Zahi Rached, Regina Albanese Pose, Érika Leão Ajala Caetano, Joana Garrossino Magalhães, Denise Grotto

**Affiliations:** 1 Toxicological Research Laboratory—Lapetox, University of Sorocaba, São Paulo 18023-000, Brazil; rana.rached@prof.uniso.br (R.Z.R.); erikacaetano01@hotmail.com (É.L.A.C.); joanagmagalhaes10@gmail.com (J.G.M.); 2 School of Health—Veterinary Medicine, University of São Caetano do Sul, São Paulo 09521-160, Brazil; regina.pose@online.uscs.edu.br

**Keywords:** antimicrobial resistance, veterinary prescriptions, companion animals, prescription surveillance, drug regulation

## Abstract

Antimicrobial resistance (AMR) is a growing global concern, influenced by antibiotic use in both human and veterinary medicine, especially in companion animals. In low- and middle-income countries, regulatory oversight on veterinary prescriptions is often limited, creating gaps that can accelerate AMR. This study aimed to characterize the use of antibiotics approved for human use that are prescribed by veterinarians for companion animals in Brazil, a country representative of broader regulatory challenges. We conducted a retrospective analysis of five years (2017–2021) of national sales data recorded by the National System for the Management of Controlled Products (SNGPC), maintained by the Brazilian Health Regulatory Agency (ANVISA). A total of 789,893 veterinary antibiotic prescriptions were analyzed over the five-year period, providing a comprehensive overview of prescribing patterns. The dataset included all oral and injectable antibiotics purchased in human pharmacies with veterinary prescriptions. Data wrangling and cleaning procedures were applied to extract information on volume, antibiotic classes, seasonal variation, and regional distribution. The results revealed a predominance of penicillins, first- and second-generation cephalosporins, and a marked increase in macrolide use, especially azithromycin. Notable regional disparities were observed, with the southeastern region leading in prescription volume. The findings, particularly the disproportionate use of azithromycin and the marked regional disparities, highlight the need for targeted monitoring policies and a stricter regulation of off-label antibiotic use in veterinary medicine. They also offer insights applicable to other countries facing similar AMR threats due to limited surveillance and regulatory frameworks.

## Simple Summary

The use of antibiotics in animals, especially pets, has raised important concerns about the development of antibiotic resistance when bacteria no longer respond to treatments that once worked. This situation affects not only animal health but also poses risks to humans. In many countries with limited resources, it is possible for pet owners to buy antibiotics in pharmacies with a simple prescription from a veterinarian, sometimes without proper guidance or follow-up. In this study, we analyzed data from pharmacies in Brazil to understand which antibiotics are being prescribed by veterinarians and how often. We focused on human-approved antibiotics prescribed for use in companion animals. Over a five-year period, we found that certain antibiotics, like penicillin, cephalosporins, and especially azithromycin, are being used more and more. Some regions of the country showed much higher use than others. For example, the Southeast region led with 9.27 prescriptions per veterinarian, followed by the Northeast with 8.6 and the South with 7.94, highlighting significant regional disparities in antibiotic use. These patterns raise important questions about access, regulation, and training. Our findings show the urgent need for better rules and education to ensure that antibiotics are used responsibly. Protecting the effectiveness of antibiotics is essential for both people and animals. This study helps demonstrate how a lack of control can contribute to a growing global health problem and highlights the importance of responsible use in veterinary care.

## 1. Introduction

Antimicrobial resistance (AMR) is a pressing global concern, driven in part by the inappropriate and unregulated use of antibiotics in both human and companion animal veterinary medicine. This study focuses exclusively on companion animals, specifically dogs and cats, which represent over 95% of the caseload in small-animal veterinary practice in Brazil. Ensuring the rational use of these substances depends not only on professional education but also on robust and transparent national regulatory systems [1,2].

The prescription and regulation of veterinary antibiotics differ markedly across geopolitical and economic contexts. In high-income countries such as the United Kingdom, regulations have been in place for a longer period and are well established. In contrast, in the United States, stricter controls were implemented more recently. For instance, the U.S. Food and Drug Administration (FDA) only mandated veterinary prescriptions for medically important antibiotics for all animal species, including livestock, starting in 2017 with the Veterinary Feed Directive (VFD), which was further reinforced in 2023 [3], whereas in the UK, despite the growing use of antimicrobials in companion animals, prescription tracking and oversight remain limited [4]. These examples illustrate the variability in regulatory maturity and the enforcement of prudent practices in high-income settings.

Conversely, regulatory challenges are even more pronounced in low- and middle-income countries (LMICs), where enforcement capacity is frequently limited. For instance, studies from Kenya report that 100% of veterinary pharmacies sell antibiotics without requiring prescriptions [5]. Similarly, in Vietnam, antimicrobials intended for veterinary use can be legally purchased without a prescription in approximately 12,000 veterinary drug shops [6]. This pattern reflects a widespread problem across LMICs, where weak regulatory enforcement, fragmented oversight systems, and high demand for antimicrobials in animal health contribute significantly to inappropriate use.

Brazil exemplifies these regulatory gaps commonly seen in LMICs. In the Brazilian context, medications intended for veterinary use fall under the jurisdiction of the Ministry of Agriculture and Livestock (MAPA), whereas antibiotics intended for human use are regulated by the National Health Surveillance Agency (ANVISA) of the Ministry of Health. Although ANVISA permits the dispensing of antibiotics approved for human use under veterinary prescription [7], MAPA’s regulations, specifically Normative Instruction No. 26 [8], do not universally mandate prescriptions for veterinary drugs. Prescription requirements are generally restricted to certain antimicrobials used as zootechnical additives in livestock production. This regulatory gap enables the widespread, and often uncontrolled, sale of antimicrobials for veterinary purposes, particularly in the care of companion animals.

Often, there is limited enforcement of prescription requirements, and over-the-counter sales of antibiotics—intended for either human or veterinary use—are commonplace. In some settings, veterinary drugs are under the jurisdiction of agricultural ministries, while human-use drugs fall under health authorities, creating fragmented oversight systems. This division may lead to inconsistencies in prescription policies and facilitate the misuse of antimicrobials in animals, particularly in the care of companion animals.

These regulatory gaps are deeply rooted in a combination of factors. Politically, the fragmented responsibilities between health (ANVISA) and agricultural (MAPA) authorities hinder the development of unified surveillance systems. Economically, limited investment in veterinary public health infrastructure reduces the capacity for enforcement and monitoring [9]. Culturally, informal practices such as over-the-counter antibiotic sales and self-medication in animal care are widespread, reflecting a lack of awareness and educational outreach regarding antimicrobial stewardship [2]

This lack of regulation, combined with the increasing demand for antibiotics to treat various conditions in pets, contributes to their indiscriminate use. Despite recommendations from veterinary associations promoting the prudent use of antimicrobials, adherence to these guidelines is often inconsistent [4]. The over-the-counter availability of these drugs, frequently without veterinary oversight, exacerbates the risk of AMR emergence in both animal and human populations.

Within the One Health framework, AMR is a critical and urgent issue that requires coordinated action across sectors—including public health, veterinary practice, pharmaceutical manufacturing, and environmental surveillance [10,11]. Thus, this study aimed to contribute to this integrated perspective by critically evaluating the use of antibiotics approved for human use in companion animals in Brazil—representing LMICs—through a retrospective, population-level analysis. Specifically, we examined veterinary prescriptions dispensed in human pharmacies, developed a national consumption profile by antibiotic class and route of administration, and analyzed regional disparities in prescription patterns. Additionally, we normalized prescription volume by the number of veterinarians practicing small-animal medicine to better understand potential misuse or overprescription.

## 2. Materials and Methods

### 2.1. Study Design

This study is a retrospective time-series analysis covering the period from 2017 to 2021. It investigates trends in the number of veterinary prescriptions for antibiotics approved for human use, the most commonly used antibiotic classes, and the regional distribution of prescriptions in Brazil. This study focuses specifically on antibiotics for oral and injectable use, as recorded in human pharmacies.

Data were sourced from the National System for the Management of Controlled Products (Sistema Nacional de Gerenciamento de Produtos Controlados, SNGPC), maintained by the Brazilian National Health Surveillance Agency (ANVISA). The decision to use ANVISA data was based on the agency’s rigorous standards for tracking antimicrobial sales. According to Collegiate Board Resolution (RDC) No. 22 of 29 April 2014 [12], pharmacies are required to retain copies of prescriptions and report sales electronically to the SNGPC. This system includes prescriptions issued by veterinarians registered with the Regional Veterinary Medicine Councils. While the MAPA regulates veterinary drugs, it lacks a centralized and digital prescription-tracking system comparable to the SNGPC. Therefore, using ANVISA data offers a more detailed and standardized source for evaluating antimicrobial use in companion animals.

It is important to note that the SNGPC database captures antimicrobial sales dispensed through human pharmacies, including prescriptions issued by veterinarians. However, it does not include sales from veterinary clinics, agro-veterinary establishments regulated by the Ministry of Agriculture (MAPA), pet shops, or online platforms authorized to sell veterinary medicines. Thus, this dataset only represents a portion of the total antimicrobial use in companion animals in Brazil, potentially underestimating the actual consumption. Due to the nature of the SNGPC database, information related to animal age, breed, or clinical condition is not available; therefore, our analysis is restricted to prescription patterns regardless of these variables.

It is important to note that in November 2021, the Brazilian Ministry of Health suspended Articles 3 and 4 of RDC 22/2014, interrupting data reporting to the SNGPC [13]. As a result, data from November 2021 onward became incomplete, with no data for December 2021 and minimal records in 2022 (less than 10% of the previous year’s average). Data for 2023 were not available at the time of submission. Consequently, our study period was defined as January 2017 to October 2021.

### 2.2. Data Extraction and Preprocessing

Monthly datasets were downloaded from the ANVISA/SNGPC portal in CSV format, corresponding to industrialized medicine sales. A total of 59 monthly prescription datasets were collected (12 months per year for 5 years, excluding December 2021).

Due to the volume of records exceeding Excel’s native row limits (1,048,576 rows), data wrangling was performed using Power Query^®^ within Microsoft Excel^®^. Files were processed and separated by year to accommodate size limitations. Records were filtered to retain only those issued by veterinary professionals and those containing antibiotics as active ingredients. Additional cleaning steps excluded records for antibiotics administered via non-oral and non-injectable routes.

To address inconsistencies and known anomalies in the ANVISA database, three outlier cases were removed:

- A single sales record of 798,365 bottles of amoxicillin–clavulanate suspension (Jardim Silva, RJ).
- A sale of 117,187 boxes of amoxicillin, clarithromycin, and lansoprazole (Rio de Janeiro, December 2018).
- A sale of 17,802 boxes of amoxicillin (Belém, Pará, July 2020).

### 2.3. Inclusion and Exclusion Criteria

This study included all medication records of molecules pharmacologically classified as antibiotics with systemic activity, dispensed through human pharmacies. When uncertainties arose regarding specific drug names or formulations, pharmacology textbooks were consulted to ensure accurate classification. Only antibiotics intended for oral (per os, PO) use or injectable use when available in human pharmacies were retained, considering their systemic action and relevance to discussions of antimicrobial resistance (AMR), particularly due to their excretion pathways. It is important to note that antibiotics commonly administered via subcutaneous (SC), intramuscular (IM), or intravenous (IV) routes within veterinary clinical settings are generally not dispensed to clients but applied directly by veterinarians or purchased through veterinary channels. Therefore, these routes are not fully represented in this dataset, which predominantly reflects oral antibiotics and the limited number of injectable formulations suitable for at-home use.

Medications administered through non-systemic routes were excluded to improve the specificity of the analysis. These included formulations intended for topical, ophthalmic, otologic, buccal, vaginal, and nasal use. The lack of standardization in how these routes were abbreviated in the prescription and sales records posed a significant data-cleaning challenge. To address this, a multi-step filtering and normalization process was implemented.

For topical use, the following abbreviations were identified and excluded: “derm,” “crem derm,” “pom derm,” “sun top,” “top powder,” “gel ct,” and “pom ct.”

Additionally, all formulations intended for dermatological, otic, ophthalmic, buccal, vaginal, nasal, and perianal use were systematically excluded from the analysis. Our study focused exclusively on antibiotics intended for systemic administration, specifically oral and injectable formulations, given their greater relevance to antimicrobial resistance dynamics. Ophthalmic medications were labeled as “oft,” “sol ocu,” and “sol oft.” Otologic routes were recorded as “sol oto,” “sus otol,” and “sus oto.” Buccal use appeared as “buccal” and “sol buc.” Vaginal formulations included labels such as “gel vag,” “gele vag,” “ovl vag,” “gynec,” and “pom vag.” Finally, nasal preparations were listed as “sol nas” and “nasal.” Abbreviations such as “sun,” “top,” “gel,” and “oto” were particularly ambiguous, as they could apply to more than one route, thus requiring broader exclusion rules.

To mitigate these inconsistencies, we applied a manual and programmatic standardization protocol using Power Query^®^. This ensured that all included data referred exclusively to antibiotics with systemic use via oral or injectable routes, thereby enhancing the accuracy and reliability of this study’s conclusions regarding antibiotic usage patterns in veterinary practice.

It is important to note that the SNGPC database only provides the number of prescriptions and units sold but does not capture the total quantity of active pharmaceutical ingredients dispensed (e.g., milligrams or kilograms). Therefore, this study does not allow the calculation of antibiotic exposure per animal based on tonnage or mg/kg metrics, representing a restriction in fully assessing the antibiotic burden on the animal population. Future studies integrating data on dosage and treatment duration are essential for a more comprehensive understanding of antimicrobial exposure in companion animals.

### 2.4. Data Organization

In the data wrangling phase (data wrangling refers to the process of cleaning raw data by identifying and resolving errors, addressing inconsistencies, and organizing variables into a usable format for analysis. No missing or incomplete values were supplemented in this study; the analysis was conducted solely based on the available dataset extracted from the SNGPC. This process is essential to ensure the integrity and usability of complex datasets (Harvard Business School Online, 2021): https://online.hbs.edu/blog/post/data-wrangling (accessed on 2 October 2023)). The dataset was systematically structured to enable consistent and accurate analysis. Antibiotic-containing products were initially classified by pharmacological class: aminoglycosides, amphenicols, carbapenems, cephalosporins, lincosamides, macrolides, nitroimidazoles, penicillins, quinolones, sulfonamides, and tetracyclines.

Subsequently, each annual file was aggregated by key analytical attributes, including the following: the total number of prescriptions issued by veterinarians; the total number of units sold (noting that some prescriptions comprised multiple packages); and the number of prescriptions assigned to each antibiotic class. These data were also organized by month, year, and federative unit (state) of origin. This step ensured the uniform structure of the database and supported subsequent exploratory analyses and the graphical representation of antibiotic prescription patterns across time and geography in Brazil.

### 2.5. Exploratory Data Analysis and Data Visualization

Following data wrangling and organization, Exploratory Data Analysis (EDA) was conducted to obtain an initial understanding of patterns, trends, and anomalies within the dataset. Although traditionally considered a preliminary step, in this study, EDA was employed iteratively and cyclically in close association with data cleaning procedures, allowing the identification of inconsistencies and the refinement of filters during processing.

The primary objective of EDA was to generate descriptive insights into veterinary prescription practices involving antibiotics approved for human use over time and across regions. To achieve this, the analysis employed frequency distributions and summary statistics to quantify prescription volume, antibiotic classes used, and monthly trends. These descriptive measures supported hypothesis generation and guided a deeper contextual interpretation of the findings.

In addition to numerical summaries, data visualization techniques were applied to illustrate temporal fluctuations and potential seasonal patterns in antibiotic use. Monthly and yearly plots of total prescriptions and antibiotic sales volume allowed for the identification of deviations, peaks, or reductions possibly linked to public health events (e.g., the COVID-19 pandemic) or regulatory changes (e.g., suspension of SNGPC data reporting in late 2021).

The combination of tabular analysis and visual representation provided a more intuitive and comprehensive understanding of the dataset. This approach facilitated the identification of regional differences and helped highlight critical points of concern in veterinary antimicrobial use within Brazil, insights that may have broader relevance for other low- and middle-income countries facing similar regulatory challenges.

### 2.6. Statistical Analysis

Data were expressed as monthly or annual means. Comparisons among years were performed using one-way ANOVA, followed by Tukey’s Honest Significant Difference (HSD) test for multiple comparisons. Statistical significance was set at *p* < 0.05. All analyses were conducted using Statistica^®^ version 8 (StatSoft Inc., Tulsa, OK, USA).

## 3. Results

The initial findings were derived from the analysis of veterinary prescriptions for antibiotics approved for human use dispensed in private pharmacies across Brazil between 2017 and 2021. In addition to antibiotics, prescriptions issued by veterinarians also included other drug classes controlled by ANVISA, such as antidepressants, anxiolytics, opioids, dopamine antagonists, anticonvulsants, antidiabetics, diuretics, and amphetamines. When all prescriptions issued by veterinarians and filled in pharmacies were aggregated (Table 1), the data revealed a consistent upward trend over the five-year period. Prescription volumes in 2019, 2020, and 2021 were significantly higher compared to 2017 (*p* = 0.0497, *p* = 0.00013, and *p* = 0.00013, respectively), indicating that the growth in prescriptions became statistically evident starting in 2019. The most substantial increase occurred between 2019 and 2020 (*p* = 0.00926).

When isolating antibiotic prescriptions from the broader dataset, similar trends were observed (Table 2). Antibiotics accounted for approximately 70% of all prescriptions issued by veterinarians, highlighting a remarkably high proportion. Prescription volumes showed a progressive increase over the years, with 2020 and 2021 being significantly higher compared to 2017 (*p* = 0.000161 and *p* = 0.000544) and 2018 (*p* = 0.001354 and *p* = 0.013044).

The most substantial annual growth occurred between 2019 and 2020, with a 12% increase, although this difference was marginally non-significant (*p* = 0.0667). Monthly peaks were particularly notable between July 2019 and July 2020 (17%) and March 2020 and March 2021 (18%), reflecting the impact of the COVID-19 pandemic on veterinary prescribing patterns.

Although the absolute number of prescriptions in 2021 was slightly lower than in 2020 mainly due to missing data for December, the monthly average remained 2% higher. No significant difference was observed between 2020 and 2021 (*p* = 0.959), indicating that the elevated prescription levels achieved during the pandemic were sustained.

**Figure 1:**
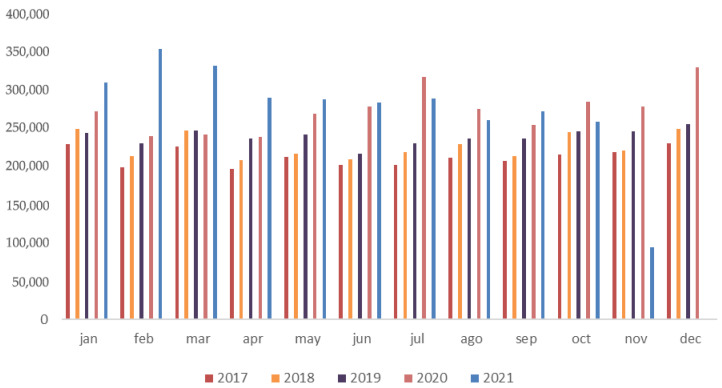
Monthly totals of antibiotic units approved for human use (boxes, vials, or packages), prescribed by veterinarians and purchased in pharmacies intended for human use in Brazil between 2017 and 2021. Note: December 2021 data are unavailable due to an interruption in SNGPC reporting.

Figure 1 displays the total number of antibiotic units (boxes, vials, or packages) prescribed by veterinarians and purchased in human pharmacies in Brazil from 2017 to 2021, on a monthly basis. The descriptive analysis shows progressive increases of 4.46%, 5.29%, and 9.25% in monthly totals between 2017 and 2018, 2018 and 2019, and 2019 and 2020, respectively, followed by a reduction of 11.76% from 2020 to 2021, which corroborates the observed decline in the second half of 2021.

The most pronounced increases were observed in June, July, and December 2020, likely associated with the impact of the COVID-19 pandemic on veterinary clinical routines. Over the five-year period, there was a cumulative mean monthly increase of 24.64% when comparing 2017 to 2021. Annual totals were 2,554,757 units in 2017; 2,723,921 in 2018; 2,870,350 in 2019; 3,280,243 in 2020; and 3,032,058 in 2021, representing increases of 6.2%, 5.1%, and 12.5%, followed by a decrease of 8.2% in 2021.

These monthly variations are presented as descriptive trends, with statistical significance testing applied exclusively to annual mean values, as detailed in the Section 3.

After applying the inclusion criteria to exclude non-oral and non-injectable formulations, the dataset was reduced by 35%, with oral and injectable antibiotics representing approximately 65% of total prescriptions. Although the number of prescriptions ranged from 40,000 to 62,000 monthly, individual records could include single or multiple units, with some reflecting bulk purchases for clinical stock.

**Figure 2:**
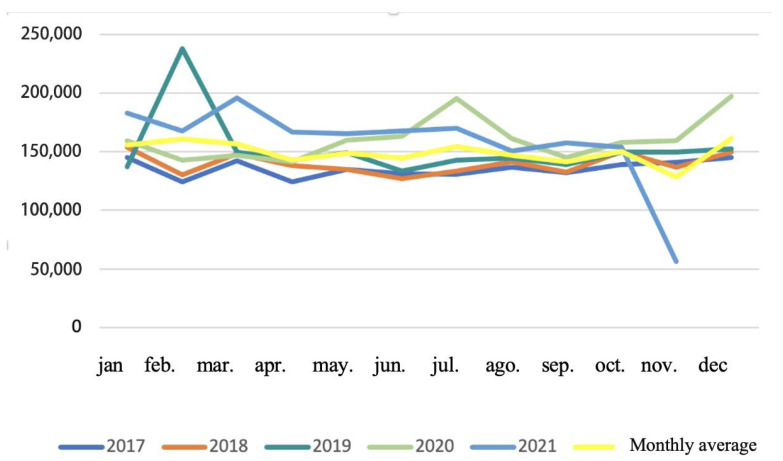
Monthly amount of oral and injectable antibiotics intended for human use, prescribed by veterinarians, and purchased in pharmacies in Brazil from 2017 to 2021.

Monthly trends in oral and injectable antibiotic sales showed moderate variability over time (Figure 2). Notably, February consistently displayed higher variability across years, and significant peaks were observed in July and December 2020, likely associated with the impacts of the COVID-19 pandemic. Despite the database interruption in December 2021 and the unusually low figure for November 2021, the overall trend indicates that prescription activity remained stable over time. These patterns are presented as descriptive trends, with statistical significance testing applied only to annual mean values, as described in the Section 3.

**Figure 3:**
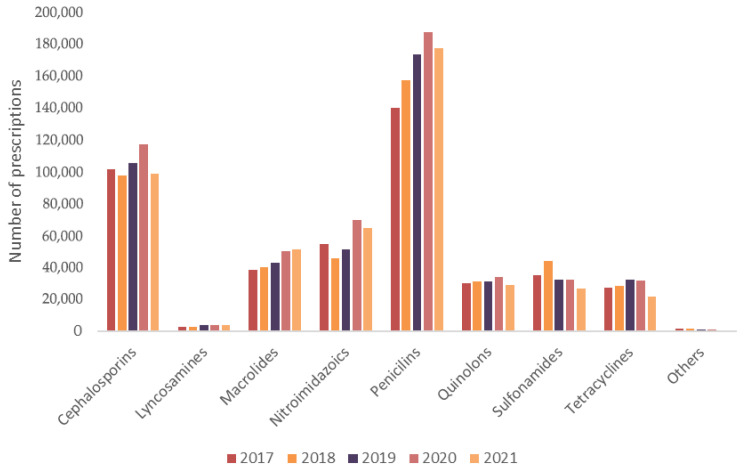
Total annual number of veterinary prescriptions for oral and injectable antibiotics intended for human use and dispensed in pharmacies in Brazil from 2017 to 2021 (categorized by antibiotic class). Note: The category “Others” includes aminoglycosides, carbapenems, amphenicols, and penicillins combined with macrolides or quinolones.

An analysis by antibiotic class revealed that penicillins (35.67%) and cephalosporins (22.08%) dominated prescription volumes, followed by nitroimidazoles (12.28%) and macrolides (9.54%). Quinolones (6.41%), sulfonamides (7.20%), and tetracyclines (5.86%) were also frequently prescribed. Less common classes included lincosamides (0.73%), amphenicols and penicillin combinations (0.10%), aminoglycosides (0.05%), and carbapenems (*n* = 10), all grouped under the “Other” category in Figure 3.

Trends over time showed a consistent increase in the use of penicillins and a notable rise in macrolide prescriptions—particularly azithromycin—during 2020 and 2021. Amoxicillin accounted for nearly 95% of all penicillin prescriptions, with more than 60% including clavulanic acid. Among cephalosporins, cephalexin represented 90% of the total. Other frequently used antibiotics included metronidazole, sulfamethoxazole + trimethoprim, ciprofloxacin, and doxycycline.

**Figure 4:**
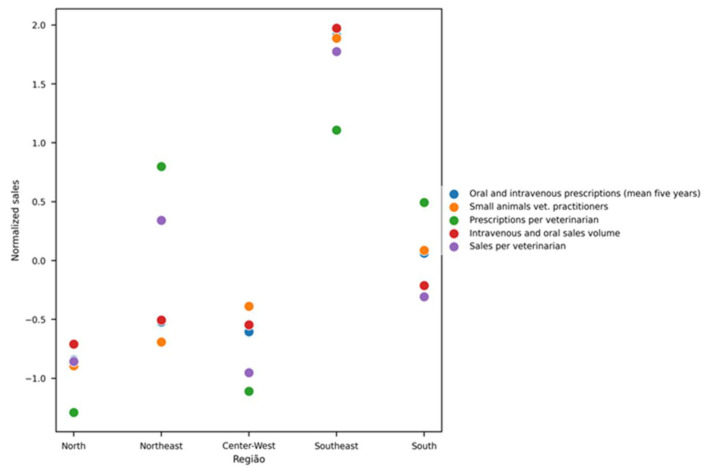
Five-year average of antibiotic-intended-for-human-use prescriptions and units sold (oral or injectable) per small-animal veterinarian, stratified in pharmacies by region of Brazil. Note: Demographic data were normalized using regional veterinary workforce demographics [14].

When analyzed by geographic region, the Southeast presented the highest rate of antibiotic prescriptions normalized by the number of veterinarians in small-animal practice (9.27 prescriptions per veterinarian), followed by the Northeast (8.6), South (7.94), Central-West (4.48), and North (4.09). In terms of total antibiotic units sold per veterinarian, the Southeast again led (38.27 units), followed by the Northeast (25.70), South (20.00), North (15.16), and Central-West (14.33) (Figure 4).

These normalized results align with regional demographic data of veterinary professionals [14], showing a higher concentration of veterinarians in the Southeast. However, an unexpected finding was the high number of prescriptions and antibiotic units sold in the Northeast, despite its relatively lower number of professionals in small-animal practice. This discrepancy suggests regional differences in access, prescription behavior, or drug sourcing practices that merit further investigation.

## 4. Discussion

Antimicrobial resistance (AMR) is a global health challenge, exacerbated by inappropriate antibiotic use in both human and veterinary medicine. While high-income countries have established regulations and surveillance systems, many low- and middle-income countries (LMICs) still face regulatory gaps and insufficient professional training, leading to the widespread and poorly controlled use of critical antimicrobials [1,2].

Brazil represents a clear example of this scenario. Although human-use antibiotics are monitored through ANVISA’s SNGPC system, veterinary antimicrobials lack a unified prescription-monitoring policy. MAPA does not require veterinary prescriptions for all antimicrobial classes, and no classes are explicitly prohibited for veterinary use. In contrast, the European Union has adopted WHO categorizations to restrict the veterinary use of antibiotics critical to human health [15], highlighting a regulatory disparity that may directly influence resistance patterns.

Our findings show a predominant use of penicillins, cephalosporins, and macrolides in companion animals, classes that are commonly used in human medicine and classified by the WHO as important or critically important [16]. Drugs such as azithromycin, ciprofloxacin, and cefuroxime, categorized as “watch group” antibiotics, were frequently prescribed [16]. The use of these classes in veterinary medicine, especially without standardized diagnostic confirmation, contributes to selective pressure and the potential transmission of resistant bacteria across species [17,18].

This trend is not exclusive to Brazil. In other LMICs, similar patterns have been documented. In Kenya, 100% of veterinary pharmacies surveyed sold antibiotics without prescriptions [5]. In Vietnam and Bangladesh, more than 45% of antibiotic dispensing sites operated without professional oversight [19]. In Latin America, a survey of online pharmacies showed that 65% of websites offered antibiotics without prescription, with high availability of fluoroquinolones, macrolides, and third-/fourth-generation cephalosporins [20].

Even in regulated systems, the overuse of broad-spectrum antibiotics persists. In Australia, 14.5% of dog consultations involved antibiotic prescriptions—38 per 1000 involving WHO high-importance drugs [21]. In Spain, nearly 40% of antimicrobials used in pets were antimicrobials approved for human use, with amoxicillin–clavulanate as the most prescribed [22]. Across Europe, penicillins, first-/second-generation cephalosporins, and macrolides remain the most used in companion animals [23], aligning with the pattern observed in our study.

The high proportion of antibiotic prescriptions in Brazil’s companion animal sector may also reflect data blind spots. Veterinary industry reports [24] suggest the declining production of veterinary antibiotics, but these figures do not include the sales of antibiotics approved for human use that were prescribed for animals. This discrepancy indicates a possible shift in procurement sources rather than an actual reduction in antimicrobial use.

It is important to note that this study does not include antibiotic sales from agro-veterinary stores, which are not captured by ANVISA’s SNGPC system. These unrecorded sales likely represent a significant portion of antimicrobial use in veterinary practice, especially in regions with limited pharmacy access. This restriction suggests that our findings may underestimate the true volume of antibiotic consumption in companion animals.

The higher prescription rates observed in the Northeast region may reflect several contributing factors. Incomplete access to veterinary-specific antibiotics in certain areas may lead veterinarians to rely more heavily on antibiotic formulations approved for human use [18]. Additionally, economic considerations, both from the perspective of pet owners seeking more affordable options and veterinarians operating in lower-income regions, may incentivize the use of widely accessible antibiotics approved for human use [2]. Furthermore, the sociocultural context, including owner expectations and informal dispensing practices, likely plays a role in this pattern.

Empirical prescribing, often performed without laboratory confirmation, remains widespread in veterinary practice. This approach is particularly evident in the frequent use of broad-spectrum antibiotics such as metronidazole and amoxicillin–clavulanate, which are commonly selected as first-line treatments for gastrointestinal and soft tissue infections in dogs and cats. This pattern, reported in Brazilian veterinary hospitals [25], aligns with our findings, suggesting that treatment decisions are frequently based on clinical judgment rather than diagnostic testing. Similarly, in Chile, only 15% of veterinarians reported requesting laboratory tests before prescribing antibiotics, highlighting a broader regional trend of reliance on empirical therapy [26]. Similar trends appear in New Zealand, South Africa, and Greece, where empirical treatment and owner influence often guide prescribing decisions [27,28].

These behaviors are influenced by gaps in veterinary education. Although Brazil’s National Curricular Guidelines recommend content on pharmacology and disease control [29], implementation varies widely. In surveys from Poland, Australia, and South Africa, veterinary students consistently expressed concern over their preparedness to prescribe antibiotics responsibly [28,30,31]. Moreover, studies show that veterinarians often feel pressure from pet owners to prescribe antibiotics unnecessarily [32,33].

It is also important to acknowledge the significant contribution of animals to biomedical research. Animal models have been essential for the development, safety assessment, and efficacy validation of antimicrobials used in both human and veterinary medicine. This contribution reinforces the One Health framework by underscoring the intrinsic interconnectedness between human, animal, and environmental health in addressing antimicrobial resistance [34].

Within the One Health framework, our findings highlight the critical need for integrated policies that address the interconnected risks of antimicrobial resistance (AMR) across human, animal, and environmental health. The patterns observed in Brazil, although geographically specific, reflect structural challenges common to many low- and middle-income countries (LMICs), in which fragmented regulatory oversight and limited surveillance compromise AMR control efforts. Addressing this issue requires coordinated actions that go beyond veterinary clinical practice, involving stringent prescription regulations, comprehensive surveillance systems that include both human and animal sectors, continuing professional education for veterinarians, and public engagement to raise awareness about the prudent use of antibiotics. This integrated approach is essential to mitigate the contribution of companion animal antimicrobial use to the broader AMR crisis.

## 5. Conclusions

This study identified a considerable number of antibiotic prescriptions for companion animals in Brazil, particularly for penicillin, cephalosporin, nitroimidazole, and macrolide classes. This finding reflects prescribing patterns that are not unlike those observed in human medicine, highlighting a broader global concern regarding antimicrobial use across species. Prescription rates were highest in the Southeast, while the Northeast presented a disproportionate number of prescriptions relative to the number of veterinarians. The lack of transparency from the veterinary pharmaceutical industry and regulatory bodies limits effective surveillance and control. Brazil’s regulatory gaps—such as the absence of mandatory prescriptions and insufficient professional training—reflect challenges common to many low- and middle-income countries.

To address antimicrobial resistance within the One Health framework, coordinated policies are needed. These must include better data integration, mandatory prescription controls, and continuing education for veterinary professionals. Strengthening stewardship practices is essential to ensuring the responsible use of antimicrobials in animal health and minimizing their impact on public health.

## Figures and Tables

**Table 1:** Monthly and annual summary for controlled substances, including antibiotics and other drug classes approved for human use, issued by veterinarians, and dispensed in pharmacies intended for human use in Brazil (2017–2021). * *p* < 0.05 in comparison to year 2017.

	2017	2018	2019	2020	2021	Monthly Average (Prescriptions)	Monthly Total (Prescriptions)
January	84,333	90,331	95,509	101,528	118,583	95,509	490,284
February	76,371	81,547	87,755	94,649	110,947	87,755	451,269
March	84,599	88,669	92,378	98,766	120,428	92,378	484,840
April	79,133	86,078	92,106	98,763	115,364	92,106	471,444
May	83,457	85,078	94,268	105,757	115,500	94,268	484,060
June	82,365	83,689	90,018	108,533	115,463	90,018	480,068
July	83,501	86,736	95,227	113,767	116,607	95,227	495,838
August	85,857	89,420	96,081	111,114	114,066	96,081	496,538
September	83,696	84,926	93,161	107,256	112,669	93,161	481,708
October	85,266	91,536	98,018	112,707	109,292	98,018	496,819
November	85,420	89,137	95,568	110,723	53,456	89,137	434,304
December	89,243	91,933	99,569	120,683	(&)	95,751	401,428
Annual average	83,603	87,423	94,138 *	107,021 *	109,307 *		
Annual total	1,003,241	1,049,080	1,129,658	1,284,246	1,202,375		

Note: This table includes the total number of prescriptions issued by veterinarians for all drug classes controlled by ANVISA, including antibiotics, antidepressants, anxiolytics, opioids, anticonvulsants, antidiabetics, dopamine antagonists, diuretics, and amphetamines, filled in human pharmacies between 2017 and 2021. (&) Data for December 2021 were not available due to the suspension of SNGPC reporting.

**Table 2:** Monthly and annual summary of antibiotics approved for human use, prescribed by veterinarians, and filled in pharmacies intended for human use in Brazil (2017–2021). * *p* < 0.05 in comparison to year 2017; ^#^ in comparison to year 2018.

	2017	2018	2019	2020	2021	Monthly Average (Prescriptions)	Monthly Total (Prescriptions)
January	60,284	64,092	65,001	71,788	75,662	65,001	336,827
February	55,313	59,810	63,188	66,658	77,127	63,188	322,096
March	60,872	66,413	67,097	68,977	84,328	67,097	347,687
April	56,565	60,422	65,629	68,945	77,341	65,629	328,902
May	59,985	62,505	67,896	73,737	74,786	67,896	338,909
June	58,970	62,282	63,595	75,250	78,356	63,595	338,453
July	66,257	63,830	66,351	79,663	67,510	66,351	343,611
August	60,648	64,303	68,016	76,322	70,964	68,016	340,253
September	59,788	61,017	65,694	68,862	75,569	65,694	330,930
October	61,215	66,786	69,906	74,716	71,715	69,906	344,338
November	61,680	60,851	68,164	73,647	36,535	61,680	300,877
December	63,317	66,455	70,637	84,238	(&)	68,546	284,647
Annual average	60,408	63,231	66,765	73,567 *^#^	71,808 *^#^		
Annual total	724,894	758,766	801,174	882,803 *^#^	789,893 *^#^		

Note: Values represent the total number of veterinary antibiotic prescriptions dispensed in human pharmacies, as recorded in the SNGPC database. “Monthly Average” refers to the average number of prescriptions per month over the five-year period. “Monthly Total” represents the cumulative sum for each month across all years. (&) Data for December 2021 were not available due to the suspension of SNGPC reporting.

## Data Availability

Data and publication materials are available from the corresponding author upon a reasonable request.

## References

[1] Ferri M., Ranucci E., Romagnoli P., Giaccone V. (2017). Antimicrobial resistance: A global emerging threat to public health systems. Crit. Rev. Food Sci. Nutr..

[2] Patel J., Harant A., Fernandes G., Ogbuabor D.C., Hoffman S.J. (2023). Measuring the global response to antimicrobial resistance, 2020–21: A systematic governance analysis of 114 countries. Lancet Infect. Dis..

[3] U.S. Food and Drug Administration (2023). FDA Fully Implements Guidance for Industry (GFI) #263, Bringing All Medically Important Antibiotics Used in Animals Under Veterinary Oversight.

[4] Hughes L.A., Williams N., Clegg P., Callaby R., Nuttall T., Coyne K., Pinchbeck G., Dawson S. (2012). Cross-sectional survey of antimicrobial prescribing patterns in UK small animal veterinary practice. Prev. Vet. Med..

[5] Muloi D., Fèvre E.M., Bettridge J., Rono R., Ong’are D., Hassell J.M., Karani M.K., Muinde P., van Bunnik B., Street A. (2019). A cross-sectional survey of practices and knowledge among antibiotic retailers in Nairobi, Kenya. J. Glob. Health.

[6] Carrique-Mas J., Van Cuong N., Truong B.D., Phu D.H., Phuc T.M., Turner H., Thwaites G., Baker S. (2019). Affordability of antimicrobials for animals and humans in Vietnam: A call to revise pricing policies. Int. J. Antimicrob. Agents.

[7] Brasil, Ministério da Saúde (1998). Portaria nº 344, de 12 de Maio de 1998. Aprova o Regulamento Técnico Sobre Substâncias e Medicamentos Sujeitos a Controle Especial.

[8] Brasil, Ministério da Agricultura, Pecuária e Abastecimento (MAPA) (2009). Instrução Normativa nº 26, de 9 de Julho de 2009. Aprova o Regulamento Técnico para a Fabricação, o Controle de Qualidade, a Comercialização e o Emprego de Produtos Antimicrobianos de Uso Veterinário.

[9] Munkholm L., Rubin O. (2020). The global governance of antimicrobial resistance: A cross-country study of alignment between the global action plan and national action plans. Glob. Health.

[10] Church N.A., McKillip J.L. (2021). Antibiotic resistance crisis: Challenges and imperatives. Biologia.

[11] Soldevila L., Valerio L., Roure S., Vallès X., Martínez-Arias A., López-Muñoz I., Pérez-Quílez O. (2023). La invasión silente de las superbacterias: Una amenaza global. Enferm. Emerg..

[12] Brasil, Ministério da Saúde (2014). Resolução de Diretoria Colegiada nº 22, de 29 de Abril de 2014. Dispõe Sobre o Sistema Nacional de Gerenciamento de Produtos Controlados—SNGPC.

[13] Agência Nacional de Vigilância Sanitária (ANVISA) (2021). Resolução da Diretoria Colegiada—RDC nº 586, de 17 de Dezembro de 2021.

[14] Wouk A.F.P.F., Martins C.M., Mondadori R.G., Pacheco M.H.S., Pinto T.G.M., Silveira M.B.G., Ferreira F. (2023). Demografia da Medicina Veterinária do Brasil 2022.

[15] Schmerold I., Van Geijlswijk I., Gehring R. (2023). European regulations on the use of antibiotics in veterinary medicine. Eur. J. Pharm. Sci..

[16] WHO (2023). The Selection and Use of Essential Medicines 2023: Web Annex C: WHO AWaRe (Access, Watch, Reserve) Classification of Antibiotics for Evaluation and Monitoring of Use.

[17] Van Boeckel T.P., Pires J., Silvester R., Zhao C., Song J., Criscuolo N.G., Gilbert M., Bonhoeffer S., Laxminarayan R. (2019). Global trends in antimicrobial resistance in animals in low- and middle-income countries. Science.

[18] Hernando-Amado S., Coque T.M., Baquero F., Martínez J.L. (2019). Defining and combating antibiotic resistance from One Health and Global Health perspectives. Nat. Microbiol.

[19] Do N.T.T., Vu H.T.L., Nguyen C.T.K., Punpuing S., Khan W.A., Gyapong M., Asante K.P., Munguambe K., Gómez-Olivé F.X., John-Langba J. (2021). Community-based antibiotic access and use in six low-income and middle-income countries: A mixed-method approach. Lancet Glob. Health.

[20] Garcia J.F., Diez M.J., Sahagun A.M., Diez R., Sierra M., Garcia J.J., Fernandez M.N. (2020). The online sale of antibiotics for veterinary use. Animals.

[21] Hur B.A., Hardefeldt L.Y., Verspoor K.M., Baldwin T., Gilkerson J.R. (2020). Describing the antimicrobial usage patterns of companion animal veterinary practices: Free text analysis of more than 4.4 million consultation records. PLoS ONE.

[22] Gómez-Poveda B., Moreno M.A. (2018). Antimicrobial Prescriptions for Dogs in the Capital of Spain. Front. Vet. Sci..

[23] European Medicines Agency (2022). Sales of Veterinary Antimicrobial Agents in 31 European Countries 2021.

[24] SINDAN (2022). Relatório Mercado de Saúde Animal: Indústria Veterinária 2021.

[25] Chicuti M.M., Paier G.G.S., Senhorello I.L.S. (2022). Avaliação do uso de antimicrobianos e suas interações medicamentosas em cães e gatos hospitalizados. Ars. Vet..

[26] Galarce N., Arriagada G., Sánchez F., Venegas V., Cornejo J., Lapierre L. (2021). Antimicrobial Use in Companion Animals: Assessing Veterinarians’ Prescription Patterns through the First National Survey in Chile. Animals.

[27] Valiakos G., Pavlidou E., Zafeiridis C., Tsokana C.N., Del Rio Vilas V.J. (2020). Antimicrobial practices among small animal veterinarians in Greece: A survey. One Health Outlook.

[28] Smith P.W., Agbaje M., LeRoux-Pullen L., Van Dyk D., Debusho L.K., Shittu A., Sirdar M.M., Fasanmi O.G., Adebowale O., Fasina F.O. (2019). Implication of the knowledge and perceptions of veterinary students of antimicrobial resistance for future prescription of antimicrobials in animal health, South Africa. J. S. Afr. Vet. Assoc..

[29] Brasil, Ministério da Educação (2019). Resolução CNE/CES nº 3/2019. Institui as Diretrizes Curriculares Nacionais do Curso de Graduação em Medicina Veterinária e dá outras providências. Diário Oficial da União.

[30] Sobierajski T., Mazińska B., Chajęcka-Wierzchowska W., Śmiałek M., Hryniewicz W. (2022). Antimicrobial and Antibiotic Resistance from the Perspective of Polish Veterinary Students: An Inter-University Study. Antibiotics.

[31] McClelland J.W., Norris J.M., Dominey-Howes D., Govendir M. (2021). Knowledge and perceptions of Australian postgraduate veterinary students prior to formal education of antimicrobial use and antimicrobial resistance. One Health.

[32] Servia-Dopazo M., Taracido-Trunk M., Figueiras A. (2021). Non-Clinical Factors Determining the Prescription of Antibiotics by Veterinarians: A Systematic Review. Antibiotics.

[33] Mateus A.L., Brodbelt D.C., Barber N., Stärk K.D. (2014). Qualitative study of factors associated with antimicrobial usage in seven small animal veterinary practices in the UK. Prev. Vet. Med..

[34] Ferdowsian H.R., Beck N. (2011). Ethical and scientific considerations regarding animal testing and research. PLoS ONE.

